# Anti-Obesity Effects of Acid-Processed *Citrus reticulata* Blanco Peel Extract Enriched in Highly Bioactive Polymethoxyflavones: Inhibition of 3T3-L1 Adipocyte Differentiation and Therapeutic Efficacy in *ob*/*ob* Mice

**DOI:** 10.3390/nu17213322

**Published:** 2025-10-22

**Authors:** Hiyoung Kim, Mi-Gi Lee, Myoung-Sook Shin

**Affiliations:** 1 School of Advanced Biotechnology, Konkuk University, Seoul 05029, Republic of Korea; reihyoung@konkuk.ac.kr; 2 Bio-Center, Gyeonggi-do Business and Science Accelerator, Suwon 16229, Republic of Korea; 3 College of Korean Medicine, Gachon University, Seongnam 13120, Republic of Korea

**Keywords:** acid hydrolysis, *Citrus reticulata* Blanco, adipogenesis, anti-obesity efficacy, 3T3-L1 preadipocytes

## Abstract

**Background/Objectives**: Chronic diseases linked to obesity represent a major global health challenge. Although pharmaceutical treatments show efficacy, their use is often limited by side effects. **Methods**: This study investigated the anti-obesity effects of acid-processed *Citrus reticulata* Blanco peels extract (CRBE) prepared through reflux extraction with 50% ethanol, followed by acid treatment using 3 M hydrogen chloride and neutralization. **Results**: Following acid treatment, the composition of the extract showed a marked increase in the 5-demethylated forms of polymethoxyflavones, particularly 5-demethylnobiletin (31.86 mg/g) and 5-demethyltangeretin (34.68 mg/g), whereas the concentrations of the typical citrus polymethoxyflavones, nobiletin (14.82 mg/g) and tangeretin (10.61 mg/g), decreased. Using 3T3-L1 preadipocytes, CRBE inhibited adipogenesis concentration dependently, substantially decreasing the expression of adipogenic transcription factors and lipid metabolism-related proteins. In *ob*/*ob* mice, oral CRBE substantially suppressed body weight gain without affecting food intake, while normalizing liver function indicators and improving serum lipid profiles by reducing total cholesterol, triglycerides, and low-density lipoprotein. **Conclusions**: Acid-processed CRBE effectively inhibits adipocyte differentiation and exhibits anti-obesity effects in vivo, offering potential as a natural agent for obesity management with minimal side effects.

## 1. Introduction

Obesity, defined as “abnormal or excessive fat accumulation that may impair health” [1], has emerged as one of the most pressing global health challenges of our time. As of 2016, 13% (650 million) of adults worldwide were classified as obese, with this prevalence continuing to rise rapidly. In South Korea, adult obesity rates reached 38.3% in 2021, representing a concerning 6.9% increase from 2011 levels [2]. Beyond its direct health implications, obesity serves as a major risk factor for numerous chronic diseases, including type 2 diabetes, cardiovascular diseases, and certain cancers, primarily through mechanisms involving insulin resistance, chronic inflammation, and increased oxidative stress [3,4].

Current pharmaceutical interventions, while demonstrating efficacy in clinical trials, face substantial limitations that restrict their widespread adoption. Available anti-obesity medications include orlistat (Xenical^®^, Alli^®^), phentermine-topiramate (Qsymia^®^), GLP-1 receptor agonists such as liraglutide (Saxenda^®^) and semaglutide (Wegovy^®^), and naltrexone-bupropion (Contrave^®^) [5,6]. However, their clinical utility is substantially hampered by adverse effects including gastrointestinal discomfort, increased heart rate, insomnia, depression, acute pancreatitis, and neuropsychiatric risks [7,8]. These safety concerns, combined with the typically single-target mechanism of synthetic drugs, highlight the urgent need for alternative therapeutic approaches.

Natural product-based interventions offer compelling advantages for obesity management due to their multi-target mechanisms and generally superior safety profiles. Plant-derived compounds containing diverse bioactive substances can simultaneously influence multiple pathways involved in obesity pathophysiology. For instance, green tea catechins promote fat oxidation while inhibiting adipocyte differentiation, and curcumin demonstrates both anti-inflammatory effects and insulin sensitivity improvement [9,10,11]. However, most existing natural product research has focused on crude extracts with variable and often insufficient concentrations of key bioactive compounds, limiting their therapeutic potential.

*Citrus reticulata* Blanco peel (tangerine peel) represents a particularly promising source of bioactive compounds for metabolic health applications. This medicinal plant, widely used in East Asian traditional medicine, contains unique polymethoxyflavones (PMFs), such as nobiletin and tangeretin, that exhibit diverse biological activities including antioxidant, anti-inflammatory, and metabolic regulatory effects [12,13]. Importantly, PMFs have demonstrated specific anti-obesity mechanisms through AMP-activated protein kinase activation, fatty acid oxidation promotion, and peroxisome proliferator-activated receptor-gamma (PPARγ) modulation [14,15,16]. A critical limitation in current citrus-based research lies in the naturally low concentrations of the most bioactive derivatives. The 5-hydroxy polymethoxyflavones, particularly 5-demethylnobiletin and 5-demethyltangeretin, typically comprise only a few percent of the total PMF content in conventional extracts. Yet numerous studies have demonstrated that these demethylated forms exhibit superior biological activities compared to their precursors, including enhanced anticancer, anti-inflammatory, and antioxidant effects, as well as improved cellular uptake [17,18,19,20,21,22]. Without specialized processing such as acid treatment, these highly bioactive compounds remain underutilized in therapeutic applications.

While previous studies have either focused on the chemical synthesis and in vitro bioactivities of highly bioactive PMF derivatives or examined whole citrus extracts without marked enrichment, few have directly demonstrated comprehensive anti-obesity effects using substantially enriched preparations. Specifically, there is limited evidence for inhibitory effects on adipocyte differentiation combined with improvements in body weight, lipid profiles, and hepatic parameters in validated animal models using acid-processed extracts with enhanced concentrations of 5-demethylated PMFs.

To address these limitations, we investigated the anti-obesity effects of acid-processed *Citrus reticulata* Blanco extract peel (CRBE) enriched in 5-demethylnobiletin and 5-demethyltangeretin using an acid hydrolysis approach. We evaluated both the inhibitory activity on adipocyte differentiation using 3T3-L1 cells and comprehensive anti-obesity efficacy in *ob*/*ob* mice, providing the first direct demonstration of enhanced therapeutic potential through targeted bioactive compound enrichment in citrus extracts.

## 2. Materials and Methods

### 2.1. Preparation of Acid-Processed CRBE

The dried peel of *Citrus reticulata* Blanco was originally obtained in January 2024 from Jeju, Korea (Human Herb, Daegu, Republic of Korea). The hydroxy polymethoxyflavone-rich extract from the peel of *Citrus reticulata* Blanco was prepared following a multi-step acid-processing method. Initially, dried tangerine peel (100 g) was subjected to extraction twice with 50% ethanol (500 mL) under reflux conditions at 50 °C for 2 h each time to obtain the crude extract (yield 22%). The extract was filtered through Whatman no. 2 filter paper, followed by filtration through a 0.45 μm membrane filter and the filtrate was collected. This filtrate was then subjected to acid hydrolysis using hydrochloric acid (3 M HCl) for 12 h to convert PMFs to hydroxylated polymethoxyflavones. After acid processing, the solution was neutralized to pH 7.0–7.2 with 1.0 M ammonium bicarbonate. The neutralized mixture was diluted to 20% ethanol (*v*/*v*) and partitioned with ethyl acetate three times at a 1:1 phase ratio, gently inverting and venting between extractions to retain inorganic salts in the aqueous phase. The combined ethyl acetate layers were collected and concentrated in vacuo, after which the material was lyophilized to dryness and accurately weighed.

### 2.2. High-Performance Liquid Chromatography (HPLC) Analysis

An HPLC analysis of the HPMF-enriched CRBE was performed using an Alliance e2690 system (Waters, Milford, MA, USA) equipped with an Alliance 996 PDA detector. The prepared organic extracts were analyzed under the following analytical conditions: a 45 min linear gradient from 5 to 100% aqueous acetonitrile containing 0.1% trifluoroacetic acid, using a Phenomenex Luna 2 C18 column (4.6 mm × 150 mm). The flow rate was maintained at 1 mL/min, with an injection volume of 10 μL (sample concentration: 1 mg/mL). The detection wavelengths were set at 215 and 325 nm. Both the column and sample compartment temperatures were maintained at 25 °C throughout the analysis.

For quantification, standard curves for each compound (nobiletin, tangeretin, 5-demethylnobiletin, and 5-demethytangeretin) were constructed using six concentrations ranging from 0.01 to 0.25 mg/mL, with corresponding HPLC peak area measurements. Identification of target compounds was based on a comparison of retention times and mass-to-charge (*m*/*z*) values with those of authentic standards.

The liquid chromatography mass spectrometry (LC–MS) analysis was performed on an Agilent 1290 Infinity HPLC system (Agilent Technologies, Santa Clara, CA, USA) coupled to a 6120 Single MS equipped with a C18 Agilent column, e.g., 2.1 × 100 mm with 1.7-μm particle size. The mobile phase consisted of solvent A (water with 0.1% formic acid) and solvent B (acetonitrile with 0.1% formic acid), and a linear gradient was applied from 5 to 100% over 15 min at a flow rate of 0.3 mL/min. The column temperature was maintained at 30 °C and the injection volume was 5 μL. MS data were acquired in positive ionization mode using electrospray ionization (ESI). Identification of target compounds was based on a comparison between mass-to-charge (*m*/*z*) values and those of the reference [23].

### 2.3. 3T3-L1 Cell Culture

The 3T3-L1 preadipocyte cell line originating from mouse tissue, which was obtained from the Korean Cell Line Bank located in Seoul, Republic of Korea, was maintained in culture using high-glucose Dulbecco’s modified Eagle medium (DMEM) supplemented with 10% bovine calf serum (BCS) for nutritional support and 1% penicillin–streptomycin antibiotic solution to prevent contamination, with incubation conditions set at 37 °C in a controlled atmosphere containing 5% carbon dioxide and appropriate humidity levels.

### 2.4. 3T3-L1 Preadipocyte Differentiation

The 3T3-L1 preadipocytes were harvested upon reaching greater than 80% confluence as an attached monolayer, beginning with removal of spent culture medium and washing with DPBS buffer. Cell dissociation was accomplished through treatment with 0.25% trypsin-EDTA for 3 min, after which cells were collected via centrifugation at 500× *g* for a 5 min duration. These harvested cells were redistributed into 96-well microplates at 1 × 10^4^ cells/well density in high-glucose DMEM containing 10% BCS and 1% antibiotics. Following a 2-day period to establish full confluence, the culture conditions were modified to high-glucose DMEM with 10% FBS and antibiotics for 48 h to induce quiescence. Adipocyte differentiation was subsequently triggered using an MDI induction cocktail consisting of IBMX (0.5 mM), insulin (10 μg/mL), and DEX (1 μM) dissolved in high-glucose DMEM with 10% FBS and penicillin–streptomycin. Test concentrations of CRBE were incorporated into the differentiation medium and administered to cells over 72 h, while atorvastatin (100 ng/mL) was employed as a positive reference treatment for an equivalent period. The differentiation protocol continued with medium exchanges every second day using insulin-supplemented DMEM (10 μg/mL insulin in 10% FBS) throughout a 6-day terminal differentiation phase.

### 2.5. 3T3-L1 Cell Cytotoxicity Analysis

Cellular toxicity evaluation of the processed CRBE treatment was performed on 3T3-L1 preadipocytes utilizing the MTT (3-[4,5-dimethylthiazol-2-yl]-2,5 diphenyl tetrazolium bromide) reduction assay as an indicator of cell survival. Initial cell seeding density was maintained at 1 × 10^4^ cells in each well of 96-well microplates using 100 μL growth medium composed of high-glucose DMEM fortified with 10% FBS, 1% penicillin–streptomycin antibiotics, and differentiation-inducing agents (comprising IBMX at 0.5 mM, insulin at 10 μg/mL, and DEX at 1 μM concentration), referred to as differentiation medium (DMI). Both CRBE samples and atorvastatin control (100 ng/mL) were dissolved in DMI before being administered to cell cultures for 72 h of exposure. After the treatment duration, each well received 10 μL of EZ-CYTOX reagent manufactured by DoGenBio (Seoul, Republic of Korea), which was incubated for one hour. Spectrophotometric measurements were taken at 450 nm, and the resulting viability data were presented as percentages normalized against untreated control cells (established as 100% survival rate).

### 2.6. Oil Red O Staining of 3T3-L1 Cells

The evaluation of intracellular lipid deposition in mature adipocytes was performed using Oil Red O staining methodology. Following the completion of adipogenic differentiation protocols, 3T3-L1 adipocytes underwent two washing steps with DPBS buffer before being treated with 10% paraformaldehyde fixative for 60 min at 4 °C to preserve cellular morphology. The fixed cells were rinsed twice with DPBS and subsequently exposed to freshly prepared Oil Red O working solution, which was formulated by combining Oil Red O stock solution with distilled water in a 3:2 proportion, equilibrating the mixture for one hour, and filtering through a 0.45-μm syringe filter to remove precipitates, with staining proceeding for 15 min. Microscopic examination revealed lipid-laden cells marked by distinctive red-colored fat droplets, indicating successful adipocyte maturation. For quantitative assessment of differentiation efficiency, the Oil Red O dye was extracted from stained cells using 100% isopropanol solvent, and the optical density of the resulting solution was determined spectrophotometrically at 500 nm wavelength.

### 2.7. Analysis of Differentiation Inhibition-Related Proteins in 3T3-L1 Cells

The evaluation of protein expression related to adipogenic differentiation and lipid metabolic pathways was performed through Western blot analysis methodology. Following experimental treatments, cells were lysed using ice-cold radioimmunoprecipitation assay buffer and maintained at 4 °C for a 30 min incubation period to ensure complete protein extraction. The lysates underwent centrifugation to obtain clear supernatants, and the resulting protein content was determined utilizing bicinchoninic acid assay kits from Bio-Rad (Hercules, CA, USA). The protein samples were then resolved through 8–10% sodium dodecyl sulfate-polyacrylamide gel electrophoresis and subsequently transferred to polyvinylidene fluoride membranes for immunodetection. The membranes underwent a blocking step for 60 min at ambient temperature using TBS-T buffer (Tris-buffered saline with 0.1% Tween 20) supplemented with 5% non-fat dry milk powder to prevent non-specific binding, followed by a 15 min washing procedure with TBS-T solution. Primary antibody incubation was performed for 4 h using antibodies specific for CCAT/enhancer-binding protein-α, PPARγ, adiponectin, and fatty acid synthase (FAS), after which horseradish peroxidase-conjugated secondary antibodies were applied for an additional hour. Following membrane washing steps, the targeted protein bands were detected and visualized through enhanced chemiluminescence reagents supplied by Amersham Life Science (Arlington Heights, IL, USA).

### 2.8. Animal Experiments

Female leptin-deficient *ob*/*ob* mice on a C57BL/6HamSlc background, aged seven weeks, were procured from the Central Lab. Animal supplier located in Seoul, Republic of Korea. The laboratory animals were kept in environmentally regulated housing facilities where ambient temperature was controlled at 23 ± 3 °C, humidity levels were maintained at 50 ± 5%, and lighting followed a 12 h light and 12 h dark cycle pattern. Throughout the study, animals received unlimited access to both food and water supplies, while physiological parameters, including body weight progression and daily food and water consumption, were recorded on a weekly basis. All procedures involving animals were performed after obtaining ethical clearance from the Institutional Animal Ethics Committee and followed the approved protocols specified in the authorization document GU1-2024-IA0010.

### 2.9. Experimental Design

**Figure 1:**
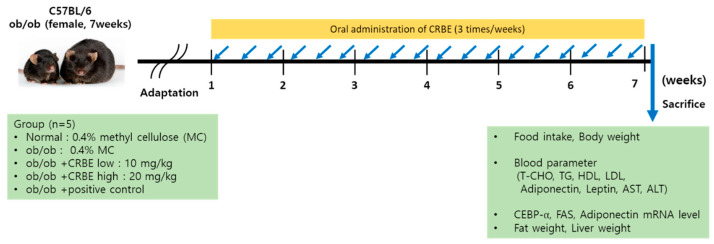
Experimental schedule for anti-obesity evaluation of the peel of Citrus reticulata Blanco extract.

After allowing the experimental animals to adapt to their new housing environment for a one-week acclimatization period, the mice were systematically distributed into experimental groups with six animals assigned to each group through the implementation of a randomized complete block experimental design to ensure unbiased group allocation. The experimental setup consisted of a normal control group composed of genetically normal wild-type C57BL/6 mice that served as healthy controls, while the obesity control group and various sample treatment groups comprised genetically modified leptin-deficient *ob*/*ob* mice that spontaneously develop obesity due to their genetic mutation, as detailed in the experimental design outlined in Table 1. For the administration protocol, all test substances were carefully prepared by creating homogeneous suspensions in a 0.5% methylcellulose vehicle solution, and these prepared formulations were then systematically administered to the designated animal groups via oral gavage three times weekly over a continuous treatment period spanning seven weeks, as illustrated in the experimental timeline shown in Figure 1. To validate the anti-obesity efficacy of the test compounds, Cissus quadrangularis extracts were incorporated into the experimental design as a positive control treatment due to their well-established anti-obesity properties and served as a reference standard for evaluating the effectiveness of the experimental treatments [24,25].

### 2.10. Biochemical Analyses of Mouse Blood

At the end of the experiment, blood was collected from the abdominal vein following a 12 h fasting period and allowed to stand at room temperature for 1 h to clot. Then, the samples were centrifuged (400× *g*, 20 min), and the serum was separated and stored at −80 °C until analysis. Biochemical parameters, including total cholesterol, triglyceride (TG), high-density lipoprotein (HDL), low-density lipoprotein (LDL), alkaline aminotransferase (ALT), and aspartate aminotransferase (AST), were determined using assay kits from Oben (Suwon, Republic of Korea).

### 2.11. Measurement of the Serum Adiponectin Content

The quantification of adiponectin levels in serum samples was performed utilizing a commercially available uncoated mouse adiponectin enzyme-linked immunosorbent assay (ELISA) kit manufactured by Elabscience (Houston, TX, USA), following a standardized protocol. The experimental procedure began with the preparation of serum samples through a 1:2 dilution process using the provided assay buffer, after which 50 μL aliquots of the diluted samples were carefully transferred into designated wells of the ELISA plate where they were left to incubate under ambient temperature conditions for a period of 2 h, with the reaction being followed by a thorough washing step repeated five times using the designated wash buffer solution. The next phase involved the addition of 50 μL of biotinylated adiponectin-specific antibody solution to each well, which was then allowed to undergo an incubation period of 1 h at room temperature to ensure adequate antibody–antigen binding. Following another series of five consecutive washing steps with the wash buffer to remove unbound components, the colorimetric detection was initiated by adding TMB substrate solution to each well, with the plates being subsequently maintained in darkness for an incubation period ranging from 5 to 10 min to allow for optimal color development. The enzymatic reaction was then halted through the addition of 50 μL of the designated stop solution to each well, and the resulting optical density values were determined by measuring the absorbance at a wavelength of 450 nm using an Emax microplate reader system (Molecular Devices, San Jose, CA, USA).

### 2.12. Evaluation of mRNA Expression of Adipogenesis-Related Genes

Frozen adipose tissue samples were thawed on ice and homogenized in RLT buffer using a disposable biomasher. Total RNA was extracted using the RNA Extraction Kit (Qiagen, Hilden, Germany) according to the manufacturer’s protocol. RNA concentration and purity were determined by measuring absorbance at 260 nm using a NanoDrop spectrophotometer (ASP-2680, ACTGene, Piscataway, NJ, USA), and only samples with an A260/280 ratio between 1.8 and 2.0 were used for downstream applications.

For cDNA synthesis, 500 ng of total RNA from each sample was used as template with the RevertAid First Strand cDNA Synthesis Kit (Thermo Fisher Scientific, Waltham, MA, USA) following the manufacturer’s instructions. The reverse transcription reaction was performed using oligo (dT) primers under the following conditions: 25 °C for 5 min, 42 °C for 60 min, followed by enzyme inactivation at 70 °C for 5 min. The synthesized cDNA was diluted with DEPC-treated water prior to subsequent analysis.

The mRNA expression levels of CCAAT-enhancer binding protein alpha (Cebpa), fatty acid synthase (Fas), and adiponectin (Adipoq) were quantified using TaqMan gene expression assays (Mm00514283_s1, Mm00662319_m1, and Mm00456425_m1, respectively), with glyceraldehyde-3-phosphate dehydrogenase (Gapdh, Mm99999915_g1) serving as the endogenous control for normalization. Real-time PCR was performed on a QuantStudio 3 system (Applied Biosystems, Waltham, MA, USA) according to the cycling conditions recommended by the TaqMan gene expression assay manufacturer. Relative gene expression was calculated using the ΔΔCt method.

### 2.13. Statistical Analyses

All values are displayed as means ± standard deviation (SD) based on three separate experimental runs. Statistical comparisons between groups were performed through one-way ANOVA and subsequent Tukey’s post hoc testing for multiple comparisons. Significance thresholds were established at *** *p* < 0.001 or **** *p* < 0.0001. Data analysis was executed using GraphPad Prism version 8.0 (GraphPad Software Inc., San Diego, CA, USA).

## 3. Results

### 3.1. Analysis of Acid-Processed CRBE

**Figure 2:**
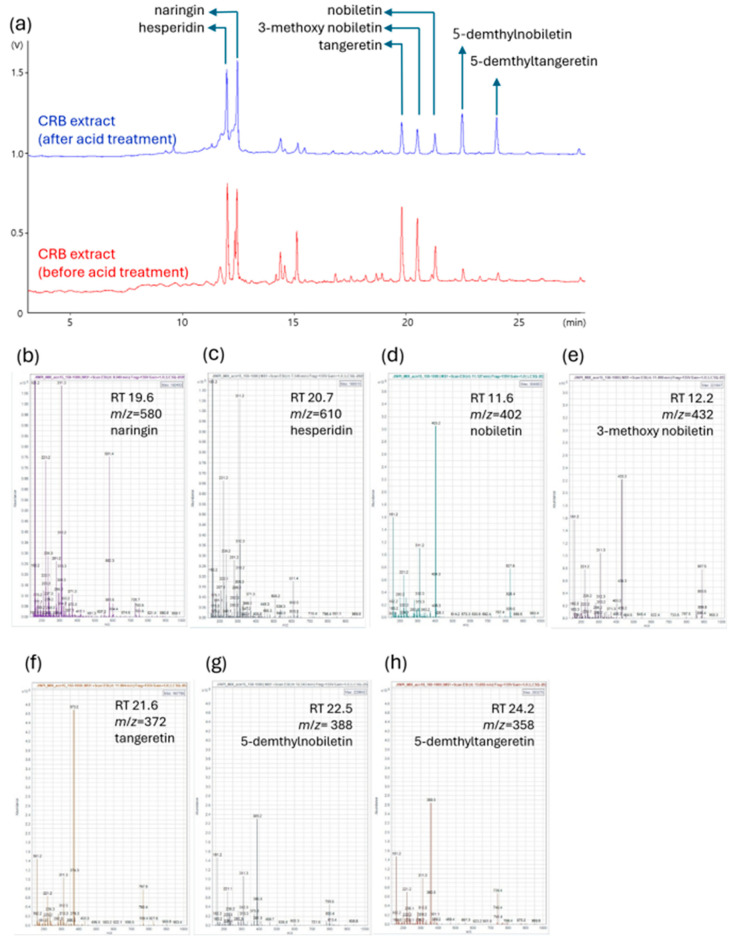
LC-MS analysis of CRB extracts before and after acid treatment. Comparison of the chromatograms illustrates the changes in flavonoid composition of the CRB extract induced by acid treatment. (**a**) Representative LC chromatograms of CRB extract before (red) and after (blue) acid treatment. Major flavonoids, naringin, hesperidin, nobiletin, 3-methoxy nobiletin, tangeretin, 5-demethyltangeretin, and 5-demethylnobiletin are annotated in the chromatogram. (**b**–**h**) Mass spectra and identification of major compounds based on retention time (RT) and mass-to-charge ratio (*m*/*z*): (**b**) naringin (RT 19.6 min, *m*/*z* = 580); (**c**) hesperidin (RT 20.7 min, *m*/*z* = 610); (**d**) nobiletin (RT 11.6 min, *m*/*z* = 402); (**e**) 3-methoxy nobiletin (RT 12.2 min, *m*/*z* = 432); (**f**) tangeretin (RT 21.6 min, *m*/*z* = 372); (**g**) 5-demethylnobiletin (RT 22.5 min, *m*/*z* = 388); (**h**) 5-demethyltangeretin (RT 24.2 min, *m*/*z* = 358).

To determine the key bioactive constituents of the acid-processed CRBE, an HPLC analysis was conducted, focusing on the quantification of the major polymethoxyflavones, nobiletin and tangeretin. CRBE was prepared by reflux extraction using 50% ethanol, followed by acid hydrolysis at pH 2.0 and subsequent neutralization. Following acid treatment and inorganic salt removal via organic solvent partitioning, the extract was recovered as a dried fraction at an overall yield of 26%. These acid treatment processes induced distinct changes in the flavonoid profile, as visualized by altered retention times and peak intensities of key components. The HPLC analysis indicated that acid-processing substantially enhanced the concentration of both 5-demethylnobiletin (31.86 mg/g) and 5-demethyltangeretin (34.68 mg/g) in the extract. The content of tangeretin was found to be 10.61 mg/g, whereas nobiletin was present at 14.82 mg/g of dried extract after the acid treatment (Figure 2 and Table 2).

### 3.2. Effect of CRBE on 3T3-L1 Cell Viability

**Figure 3:**
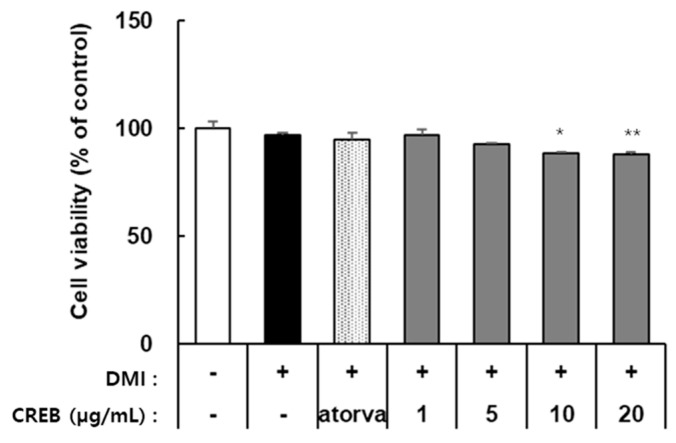
Effect of *Citrus reticulata* Blanco extract (CRBE) on the viability of 3T3-L1 cells. 3T3-L1 preadipocytes were treated with Atorvastatin (100 ng/mL), 1, 5, 10, and 20 μg/mL of CRBE in differentiation medium containing MDI (IBMX, dexamethasone, and insulin) for 72 h. Cell viability was measured by the EZ-Cytox assay. Values are expressed as percentages of control (untreated cells) and represent means ± SD from three independent experiments. Data are expressed as means ± SD from three independent experiments. Statistical significance was determined by one-way ANOVA followed by Tukey’s post hoc test. * *p* < 0.01 or ** *p* < 0.001 versus control group.

CRBE treatment decreased the viability of 3T3-L1 cells in a concentration-dependent manner. As shown in Figure 3, cell viability remained at approximately 95% and 90% at CRBE concentrations of 1 and 5 μg/mL, respectively. However, viability was significantly reduced to approximately 85% at 10 μg/mL and 83% at 20 μg/mL. Atorvastatin (100 ng/mL), used as a positive control, did not affect cell viability under the same conditions (Figure 3). Therefore, subsequent experiments to assess the inhibitory activity of CRBE on preadipocyte differentiation were conducted at 10 and 20 µg/mL.

### 3.3. Inhibitory Effect of CRBE on 3T3-L1 Cell Differentiation

**Figure 4:**
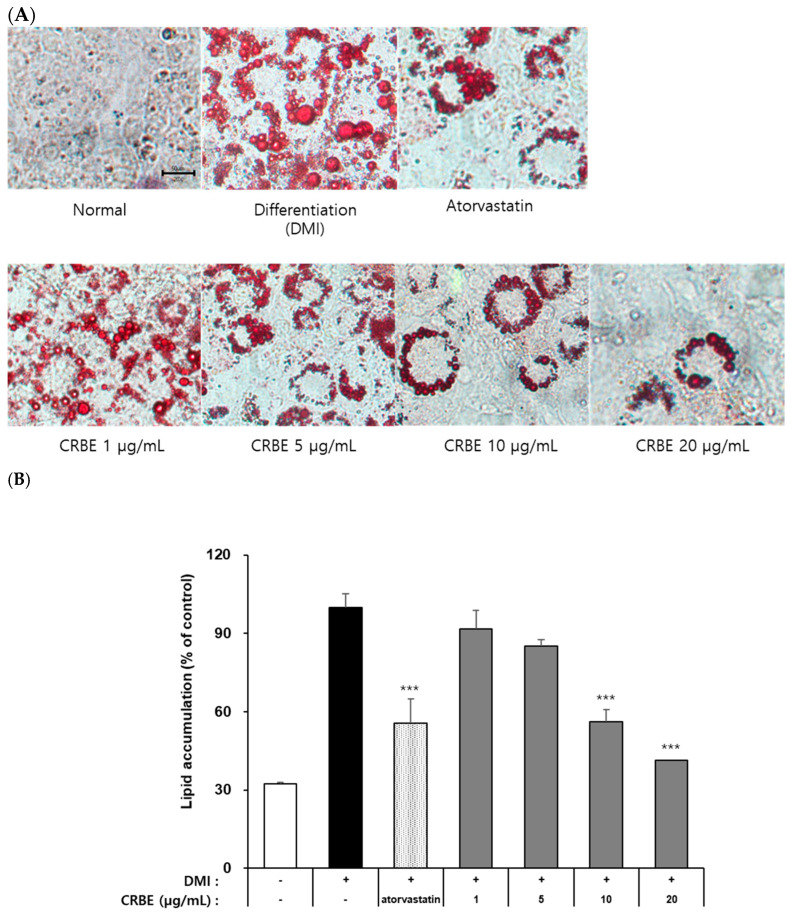
Effect of *Citrus reticulata* Blanco extract (CRBE) on lipid accumulation in 3T3-L1 adipocytes. 3T3-L1 cells were differentiated with MDI cocktail (IBMX 0.5 mM, insulin 10 μg/mL, dexamethasone 1 μM) with or without CRBE (1–20 μg/mL) for 72 h, followed by insulin treatment for 6 days. Atorvastatin (100 ng/mL) served as the positive control. (**A**) Images of Oil Red O staining of lipid droplets using a microscope. (**B**) Quantification of the lipid content by extracting Oil Red O with isopropanol and measuring the absorbance at 500 nm. Data are expressed as means ± SD from three independent experiments. Statistical significance was determined by one-way ANOVA followed by Tukey’s post hoc test. *** *p* < 0.001 versus DMI-treated group.

The 3T3-L1 preadipocytes accumulate intracellular neutral lipids (TG, lipid droplets) when treated with differentiation-inducing hormones, such as IBMX, insulin, and DEX. Evaluation of the effects of CRBE on lipid accumulation during 3T3-L1 adipocyte differentiation using Oil Red O staining indicated that cells in the differentiation-induced group (DMI-treated) exhibited marked intracellular neutral lipid accumulation compared with undifferentiated preadipocytes on day 8 (Figure 4A). Atorvastatin (100 ng/mL; positive control), which is known to suppress PPARγ and CCAT/enhancer-binding protein-α expression, substantially inhibited cell differentiation and lipid accumulation by approximately 40%. CRBE treatment at 1, 5, 10, and 20 μg/mL showed a concentration-dependent decrease in lipid accumulation (Figure 4B). Especially at 10 and 20 μg/mL, CRBE substantially inhibited lipid accumulation, similar to that of atorvastatin. A quantitative analysis by dissolving the Oil Red O contained in these cells showed that the accumulation of neutral lipids substantially decreased as the CRBE concentration increased, with 20 μg/mL CRBE treatment substantially inhibiting lipid accumulation by approximately 58% compared to the DMI-treated group.

### 3.4. Effect of CRBE on Adipogenesis-Related Proteins in 3T3-L1 Cells

**Figure 5:**
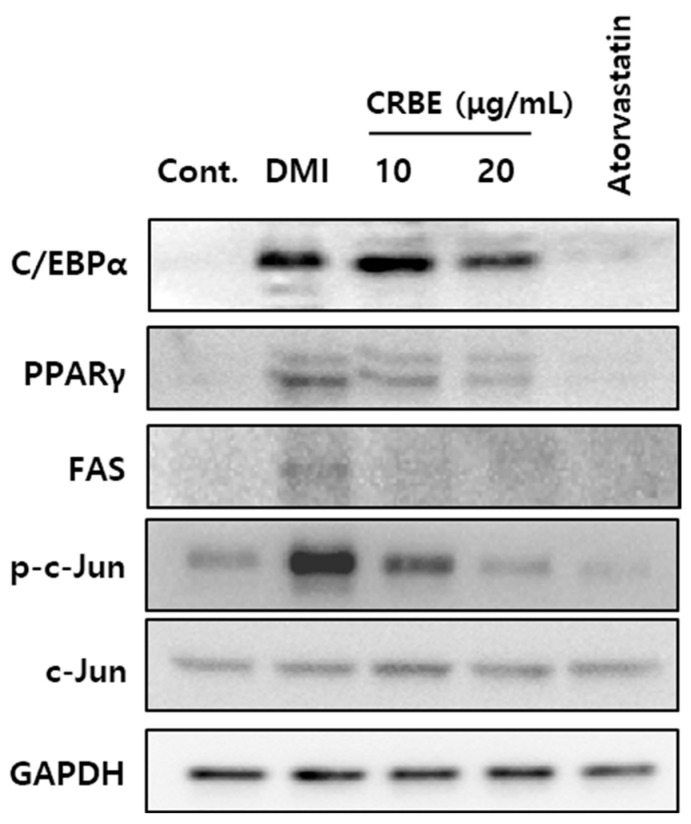
Effect of Citrus reticulata Blanco extract (CRBE) on adipogenic and lipogenic protein expression in 3T3-L1 adipocytes. 3T3-L1 preadipocytes were differentiated with MDI cocktail (IBMX 0.5 mM, insulin 10 μg/mL, dexamethasone 1 μM) in the presence or absence of CRBE (10 or 20 μg/mL) for 72 h, followed by insulin treatment for 6 days. Atorvastatin (100 ng/mL) served as the positive control. Western blot analyses of adipogenic transcription factors (CCAAT-enhancer protein alpha CEBPα, peroxisome proliferator-activated receptor gamma (PPARγ)) and lipid metabolism-related protein (fatty acid synthase, FAS), phosphorylated (p)-c-Jun, c-Jun) is shown. GAPDH was used as the loading control.

Adipocyte differentiation follows a sequential process that is regulated by various transcription factors. Figure 4 shows that the differentiation of 3T3-L1 cells with DMI substantially increased the expression of CEBPα and PPARγ, as well as FAS and adiponectin, which are involved in lipid metabolism. Treatment with CRBE (10 and 20 μg/mL) resulted in a concentration-dependent decrease in the expression of these transcription factors and lipid metabolism-related proteins, with the highest concentration (20 μg/mL) showing an inhibitory effect comparable to atorvastatin. Additionally, CRBE suppressed the phosphorylation of c-Jun, a key mitogen-activated protein kinase signaling pathway-related factor that was upregulated by DMI treatment (Figure 5).

### 3.5. Effects of CRBE on Body Weight, Food Intake, and Abdominal Fat

**Figure 6:**
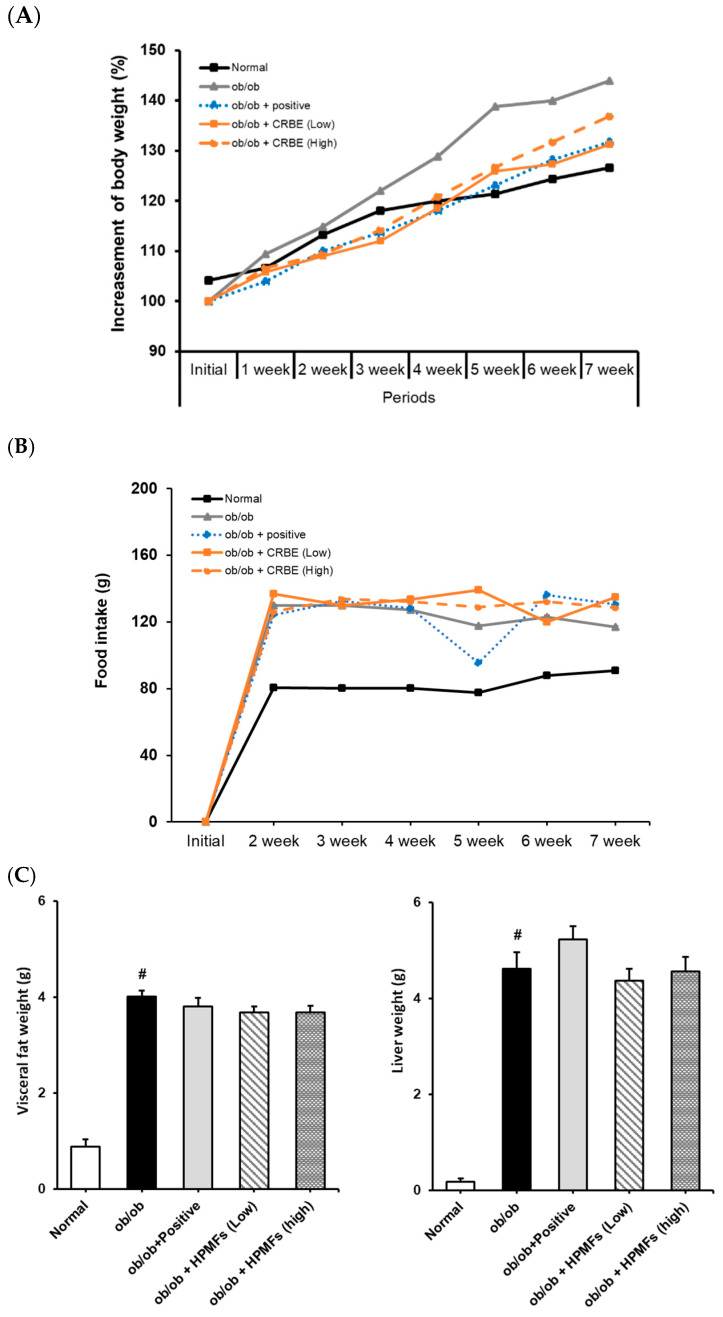
Effect of Citrus reticulata Blanco extract (CRBE) on the body weight of *ob*/*ob* mice. *ob*/*ob* Mice were administered Cissus (200 mg/kg; positive control) or CRBE (Low 10 mg/kg or High 20 mg/kg) orally three times a week for 7 weeks. The body weight (**A**) Food intake (**B**) and visceral fat and livers (**C**) were analyzed. Data are expressed as means ± SD from five independent mice (*n* = 5). Statistical significance was determined by one-way ANOVA followed by Tukey’s post hoc test. ^#^ *p* < 0.0001 compared to the normal groups.

Next, we evaluated the long-term effects of CRBE on weight gain and fat accumulation in genetically leptin receptor-deficient obese mice. An analysis of body weight changes over 7 weeks showed that the normal group exhibited a moderate weight gain (approximately 26%). In contrast, the *ob*/*ob* group showed a substantially accelerated weight gain compared to the normal group (>43% at the end of the experiment). Mice in both the positive control (*Cissus* extract), low-dose CRBE and high-dose CRBE groups showed reductions in weight gain compared to the obese model group (32%, 32% and 31%, respectively). Notably, no statistical difference was observed between the high- and low-dose CRBE groups (Figure 6A). The food intake analysis indicated no marked changes across the experimental groups following CRBE administration (Figure 6B), suggesting that the observed weight reduction was mediated by physiological mechanisms other than appetite suppression. A post-experiment analysis of abdominal adipose tissues showed substantial abdominal fat accumulation in the *ob*/*ob* control group compared to that in the normal group. In contrast, abdominal fat and liver weight showed a decreasing trend in the CRBE administration groups compared with those in the *ob*/*ob* control group (Figure 6C). Given these findings, both CRBE doses demonstrated comparable anti-obesity efficacy; however, the lower dose may be preferable for functional food applications due to advantages in cost-effectiveness and safety considerations.

### 3.6. Effects of CRBE on the Blood Lipid Profile, Adiponectin Level, and Liver Function Markers of ob/ob Mice

**Figure 7:**
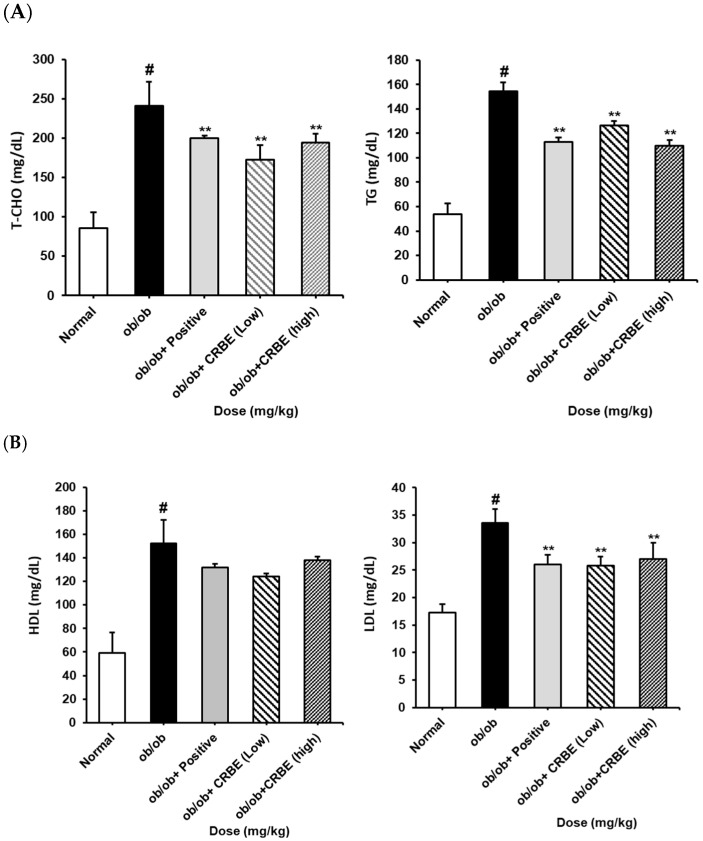

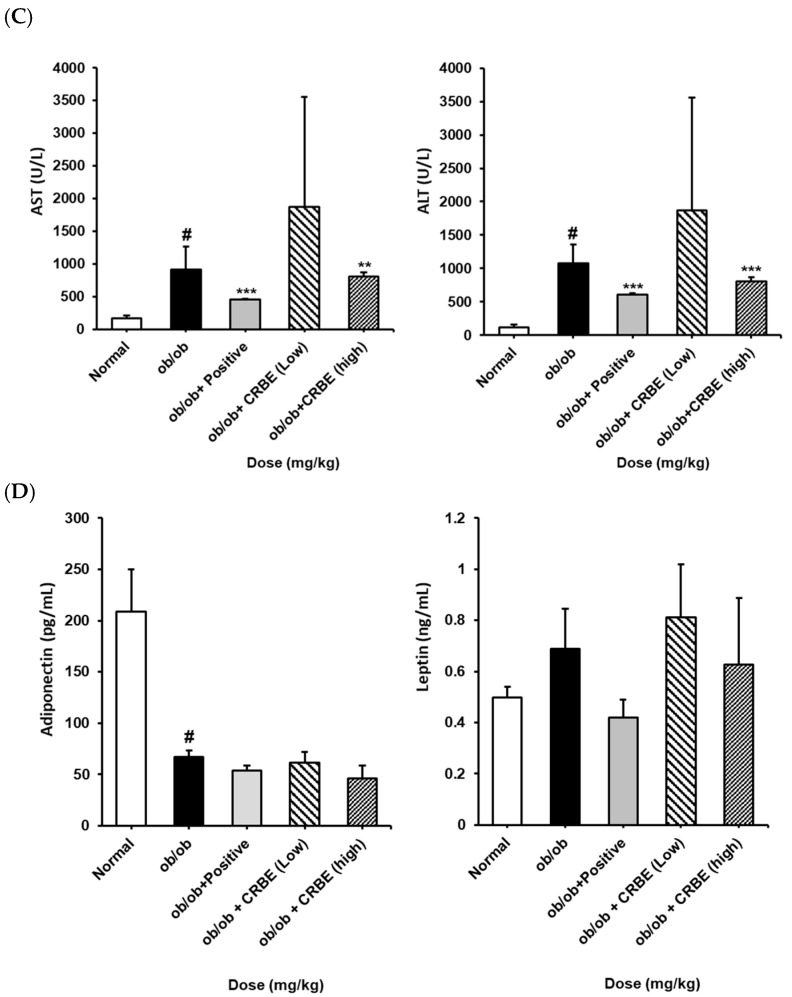
Serum biochemical values, and adiponectin and leptin levels, in *ob*/*ob* mice. CRBE low (10 mg/kg) or high (20 mg/kg) was given orally three times a week for 7 weeks. Serum was then collected and analyzed for: (**A**) total cholesterol (T-CHO) and triglycerides (TG), (**B**) high-density lipoprotein (HDL) cholesterol and low-density lipoprotein (LDL) cholesterol, (**C**) aspartate aminotransferase (AST) and alanine aminotransferase (ALT), and (**D**) adiponectin and leptin. Data are expressed as means ± SD from five independent mice (*n* = 5). Statistical significance was determined by one-way ANOVA followed by Tukey’s post hoc test. ^#^ *p* < 0.0001 compared to the normal group, ** *p* < 0.001 or *** *p* < 0.001 compared to the *ob*/*ob* group.

An analysis of the serum lipid profile markers indicated substantially elevated total cholesterol, TG, and LDL levels in the obese group. However, these parameters were markedly reduced in the groups treated with CRBE for 7 w compared with those in the obese group (Figure 7A,B). This effect was similar to that observed in the positive control group, though it was not dose-dependent. The liver function indicators, AST and ALT, were substantially increased in the obese group but substantially decreased in the positive control and high-dose CRBE groups (Figure 7C). Adiponectin levels were drastically reduced in the obese group; however, no marked recovery was observed following CRBE administration (Figure 7D).

### 3.7. Effects of Oral Administration of CRBE on the mRNA Expression of Cebpa, Fas, and Adipoq in Adipose Tissue

**Figure 8:**
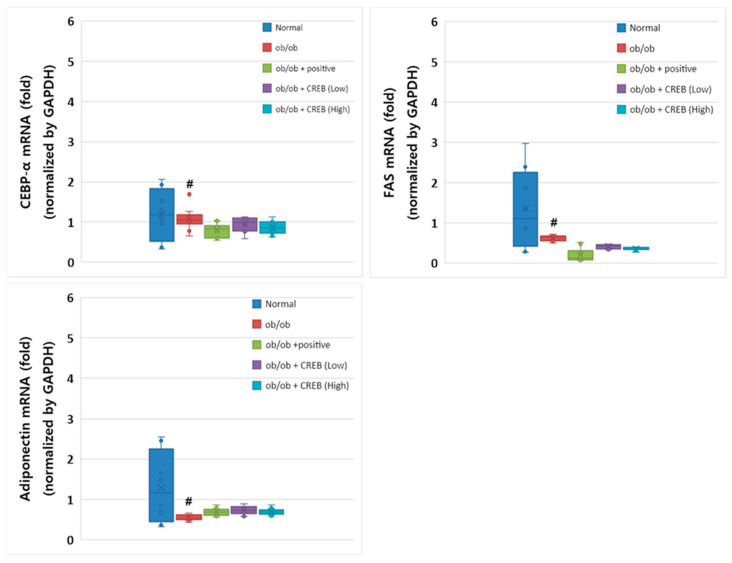
Effect of oral *Citrus reticulata* Blanco extract (CRBE) administration on the expression of *Cebpa*, *Fas*, and adiponectin (*Adipoq*) mRNA in *ob*/*ob* mice. CRBE low (10 mg/kg) or high (20 mg/kg) was given orally three times a week for 7 weeks. Adipose *Cebpa*, *Fas*, and *Adipoq* mRNA expression levels were analyzed using a real-time quantitative polymerase chain reaction system. All data are presented as means ± standard deviation (*n* = 5). ^#^ *p* < 0.0001 compared to the normal group.

Despite the genetic constraints of the leptin-deficient *ob*/*ob* mouse model, *Cebpa* mRNA expression was substantially higher in the normal group compared to that in the *ob*/*ob* control group (Figure 8). The CRBE treatment groups (10 and 20 mg/kg) showed a slight elevation in *Cebpa* expression compared to the *ob*/*ob* control group; however, these differences did not reach statistical significance. The positive control group (*Cissus* extract) showed expression patterns similar to those of the CRBE treatment groups. *Fas* mRNA expression showed a pronounced reduction in all *ob*/*ob* groups compared to that in the normal control group. In particular, both CRBE treatment doses maintained *Fas* expression at levels comparable to those in the positive control group, suggesting the potential regulation of lipid synthesis pathways despite the genetic limitations of the *ob*/*ob* model. The mRNA expression of *Adipoq*, which plays an important role in insulin sensitivity, was substantially reduced in the *ob*/*ob* control group compared to that in the normal group. The CRBE treatment groups showed modest increases in adiponectin expression compared to the control *ob*/*ob* group, with the low-dose group exhibiting slightly higher expression than the high-dose group; however, these differences were not statistically significant.

## 4. Discussion

The results of the present study demonstrate that acid-processed CRBE exhibits considerable anti-obesity effects through multiple mechanisms targeting adipocyte differentiation and lipid metabolism. The acid-processing method used (reflux with 3 M HCl for 12 h) effectively enhanced the extract composition by markedly increasing the content of bioactive hydroxypolymethoxyflavones, yielding substantial amounts of 5-demethylnobiletin and 5-demethyltangeretin. The concentrations of these compounds far exceeded those obtained by conventional extraction methods. In 3T3-L1 preadipocytes, CRBE demonstrated potent inhibition of adipogenesis (58% at 20 μg/mL) by downregulating critical transcription factors (CEBPα and PPARγ) and modulating the mitogen-activated protein kinase signaling pathway through the suppression of c-Jun phosphorylation, consistent with recent findings on PMF mechanisms of action. The in vivo efficacy of CRBE in *ob*/*ob* mice was evidenced by marked reductions in body weight gain without affecting food intake, suggesting metabolic regulation rather than appetite suppression—a notable advantage over many conventional anti-obesity agents. The pronounced improvements in serum lipid profiles (23% reduction in total cholesterol, 18% in TG, and 27% in LDL-cholesterol) and liver function markers (AST, ALT) further emphasize CRBE’s potential as a multifunctional agent addressing the metabolic syndrome cluster.

Although gene expression changes in adipose tissue were modest, possibly due to the complex leptin-deficient phenotype of the *ob*/*ob* mouse model, these results aligned with the in vitro observations and previous literature on citrus flavonoid effects [14,15,16]. Importantly, CRBE achieved these therapeutic effects at relatively low doses (10–20 mg/kg) without observable adverse effects, highlighting its potential as a safe, functional food ingredient. The synergistic activity of multiple bioactive compounds in CRBE may provide advantages over isolated compounds or single-target pharmaceuticals by simultaneously influencing various obesity-related pathways. This study adds to the growing evidence supporting natural product-based approaches for metabolic disorders, with CRBE representing a promising candidate for functional food development.

## 5. Conclusions

This study demonstrated that acid-processed *Citrus reticulata* Blanco peel extract (CRBE), enriched with 5-demethylnobiletin (31.86 mg/g) and 5-demethyltangeretin (34.68 mg/g), exhibited partial anti-obesity effects with notable limitations.

CRBE inhibited adipocyte differentiation in 3T3-L1 cells (58% at 20 μg/mL) through downregulation of CEBPα and PPARγ, though this concentration also caused 17% cytotoxicity. In *ob*/*ob* mice, CRBE (10–20 mg/kg) reduced body weight gain and improved lipid profiles without affecting food intake. However, the therapeutic potential of CRBE was limited by several key findings. Despite testing two different doses, no dose-dependent response was observed, and notably, serum adiponectin levels remained suppressed even after weight reduction. Furthermore, the expected molecular changes in adipose tissue were minimal, with most gene expression alterations failing to achieve statistical significance. The discrepancy between in vitro and in vivo results, particularly the lack of significant molecular changes in adipose tissue, indicates an incomplete understanding of CRBE’s mechanisms. Furthermore, the *ob*/*ob* mouse model’s inherent limitations due to leptin deficiency may not accurately represent human obesity.

While acid processing successfully enhanced bioactive compound concentrations, the current evidence supports only limited anti-obesity efficacy. Future studies should address the narrow therapeutic window, validate findings in diet-induced obesity models, and establish clear mechanisms of action before considering CRBE for functional food development.

## Figures and Tables

**Table 1:** Grouping and treatment of experimental mice.

Mice Type	Group	Dose (mg/kg)
C57BL/6HamSlc +/+	Normal (0.5% methylcellulose 400)	vehicle only
C57BL/6HamSlc-*ob*/*ob*	0.5% methylcellulose 400	vehicle only
	*Citrus reticulata* Blanco extract (low)	10 mg/kg
	*Citrus reticulata* Blanco extract (high)	20 mg/kg
	*Cissus* extracts (positive control)	200 mg/kg

**Table 2:** Flavonoid Content in Citrus Extract Before and After Acid Treatment.

Compound	Before Acid Treatment (mg/g)	After Acid Treatment (mg/g)
Nobiletin	44.46 ± [2.30] (*n* = 3)	24.82 ± [1.83] (*n* = 3)
Tangeretin	18.36 ± [1.31] (*n* = 3)	10.61 ± [0.54] (*n* = 3)
5-Demethylnobiletin	10.34 ± [0.89] (*n* = 3)	31.86 ± [0.41] (*n* = 3)
5-Demethyltangeretin	7.63 ± [1.15] (*n* = 3)	34.68 ± [1.28] (*n* = 3)

## Data Availability

The original contributions presented in this study are included in the article. Further inquiries can be directed to the corresponding authors.

## Abbreviations

The following abbreviations are used in this manuscript:

ALT	alkaline aminotransferase
AST	aspartate aminotransferase
BCS	bovine calf serum (BCS)
CRBE	dried peel of citrus reticulata Blanco extract
DEX	dexamethasone
DMEM	Dulbecco’s modified Eagle medium
DMI	differentiation medium
DPBS	Dulbecco’s phosphate-buffered saline
ELISA	enzyme-linked immunoassay
ESI	electrospray ionization
FAS	fatty acid synthase
FBS	fetal bovine serum
HDL	high-density lipoprotein
HPLC	high-performance liquid chromatography
HPMFs	hydroxylated polymethoxyflavones
LC–MS	liquid chromatography mass spectrometry
LDL	low-density lipoprotein
MTT	3-[4,5-dimethylthiazol-2-yl]-2,5 diphenyl tetrazolium bromide
PMFs	polymethoxyflavones
PPARγ	peroxisome proliferator-activated receptor-gamma
TBS	tris-buffered saline
TG	triglyceride
